# Cardiac Effects of Treadmill Running at Different Intensities in a Rat Model

**DOI:** 10.3389/fphys.2021.774681

**Published:** 2021-11-29

**Authors:** Zhipeng Yan, Ni Zeng, Jieting Li, Tao Liao, Guoxin Ni

**Affiliations:** ^1^Department of Rehabilitation Medicine, First Affiliated Hospital, Fujian Medical University, Fuzhou, China; ^2^Department of Rehabilitation Medicine, The Affiliated Hospital of Guizhou Medical University, Guizhou, China; ^3^Department of Rehabilitation Medicine, Fuzhou Second Affiliated Hospital, Xiamen University, Fuzhou, China

**Keywords:** exercise, treadmill running, cardiac structure and function, cardiac hypertrophy, ERK signaling pathway

## Abstract

**Purpose:** In this study, we investigated the effect of treadmill exercise training on cardiac hypertrophy, collagen deposition, echo parameters and serum levels of cardiac troponin I (cTnI) in rats, and how they differ with various exercise intensities, hence exploring potential signal transduction.

**Methods:** Male Sprague-Dawley rats were randomly divided into sedentary (SED), low-intensity running (LIR), medium-intensity running (MIR), and high-intensity running (HIR) groups. Each exercise group had 3 subgroups that were sacrificed for cardiac tissue analyses at 1, 4, and 8 weeks, respectively, and all rats participated in a daily 1 h treadmill routine 5 days per week. Echocardiographic measurements were performed 24 h after the last exercise session. Additionally, myocardium samples and blood were collected for histological and biochemical examinations. Changes in the extracellular signal-regulated kinases 1/2 (ERK1/2) signal pathway were detected by Western blotting.

**Results:** After a week of running, ventricular myocyte size and the phosphorylation of ERK1/2 increased in the HIR group, while left ventricular (LV) diastolic diameter values and LV relative wall thickness increased in the LIR and MIR groups. In addition, we observed heart enlargement, cTnI decrease, and ERK1/2 signal activation in each of the exercise groups after 4 weeks of running. However, the HIR group displayed substantial rupture and increased fibrosis in myocardial tissue. In addition, compared with the LIR and MIR groups, 8 weeks of HIR resulted in structural damage, fiber deposition, and increased cTnI. However, there was no difference in the activation of ERK1/2 signaling between the exercise and SED groups.

**Conclusion:** The effect of running on cardiac hypertrophy was intensity dependent. In contrast to LIR and MIR, the cardiac hypertrophy induced by 8 weeks of HIR was characterized by potential cardiomyocyte injury, which increased the risk of pathological development. Furthermore, the ERK signaling pathway was mainly involved in the compensatory hypertrophy process of the myocardium in the early stage of exercise and was positively correlated with exercise load. However, long-term exercise may attenuate ERK signaling activation.

## Introduction

Exercise and physical activity are effective ways to reduce the risk of cardiovascular diseases, such as heart attack and stroke, and can provide valuable benefits beyond those of medications. Improved cardiac performance, along with cardiac hypertrophy, is a major feature of endurance exercise, leading to a constellation of adaptations that affect the structure, electrical conduction, and function of the heart and contribute to appropriate increases in cardiac output (Khoury et al., 2019; Moreira et al., 2020). Studies in animal models of exercise-induced cardiac hypertrophy (here in response to treadmill exercise, voluntary wheel running, and swim training) have shown preserved or enhanced contractile function and relative cardiac hypertrophy (Konhilas et al., 2004; Radovits et al., 2013; Mi et al., 2019). The important differences that exist between these experimental methods may affect the interpretation of the results. There is a plethora of studies demonstrating that treadmill running is a preferred option in comparative studies of the effects of exercise training because of its ability to precisely control exercise intensity and volume (Kemi et al., 2004; Wang et al., 2010; Tang et al., 2011).

Physiological cardiac hypertrophy induced by endurance exercise is related to an increase in cardiac mass and individual cardiomyocyte growth in both length and width, with no interstitial or replacement fibrosis or cell damage; this is considered reversible and does not develop into heart failure (Ellison et al., 2012). In contrast, although pathological hypertrophy is initially induced as a compensatory response to the growth of the ventricle, this kind of hypertrophy progresses to ventricular chamber dilatation, with wall thinning through the loss of myocytes and contractile dysfunction, resulting in adverse cardiovascular events (Nakamura and Sadoshima, 2018). The impact of endurance exercise on heart health depends on the combination of intensity, time duration, frequency, and exercise type (Davos, 2019). However, less is known regarding cardiac adaptations to the different intensities of treadmill running in rats. Most studies have shown that low- and moderate-intensity exercise attenuates abnormal cardiac remodeling and myocardial dysfunction and improves functional capacity (Mi et al., 2019; Pagan et al., 2019). Additionally, clinical studies also support this recommendation by identifying the benefits that can be derived from low- and moderate-intensity exercise (Wasfy and Baggish, 2016). Exercise performed at a high intensity appears to convey greater cardioprotective benefits than exercise of a moderate intensity, which may be because of the increase in aerobic fitness (Swain and Franklin, 2006). However, recent data show that high-intensity exercise has an adverse effect on the heart, along with impairment of cardiomyocyte Ca^2+^ handling, mitochondrial respiration, and the activation of proapoptotic and profibrotic activity (Olah et al., 2015; Ljones et al., 2017). Given that intensity dependence for cardiac function remains controversial, it is imperative to better understand how different treadmill running intensities alter the phenotype of the murine heart.

Exploring the molecular mechanisms of cardiac hypertrophy is helpful to better understand the adaptability of the heart. Among the numerous signaling factors, the extracellular signal-regulated kinases 1/2 (ERK1/2) pathway has been suggested to promote cardiac hypertrophy (Yan et al., 2021). For instance, transgenic mice overexpressing an activated dual specificity mitogen-activated protein kinase kinase 1 (MEK1) mutant under the transcriptional control of the cardiac-specific α-myosin heavy chain promoter were found to induce cardiac hypertrophy *in vivo*, which constitutively activated ERK1/2 in the heart (Bueno et al., 2000). Furthermore, another study demonstrated that the ERK1/2 signaling pathway uniquely regulates the balance between eccentric and concentric growth of the heart (Kehat et al., 2011). Even though many studies conducted on pressure overload or mutagenesis have examined the role of the MEK1/2-ERK1/2 pathway, the regulatory mechanisms for exercise-induced cardiac hypertrophy remain unclear.

The present study was designed to develop a rat model of three treadmill running programs to test whether different types of intensity training can induce cardiac structural and functional changes. Our hypothesis was that different exercise loads would result in intensity-dependent myocardial hypertrophy, which might be associated with different functional consequences in the left ventricle (LV) in rats. Therefore, we aimed to provide a structural and functional characterization of the LV in exercise-induced hypertrophy in rats. Additionally, we investigated molecular alterations in exercise-induced cardiac hypertrophy.

## Materials and Methods

### Experimental Animals and Exercise Protocols

A total of 72 male Sprague-Dawley rats of 8 weeks of age and200–220 g in weight were randomized into four groups of the same size: (1) sedentary control (SED, *n* = 18), (2) low-intensity running (LIR, *n* = 18), (3) medium-intensity running (MIR, *n* = 18), and (4) high-intensity running (HIR, *n* = 18). Each exercise group was divided into three subgroups which were sacrificed for evaluation of cardiac tissues at 1, 4, and 8 weeks, respectively, following the end of the last training (*n* = 6 for each time subgroup). The rats were housed in cages under controlled temperature (22 ± 1°C) and, humidity (50%) conditions with a 12/12 h light/dark cycle and were allowed *ad libitum* access to standard rodent chow and water. This study was approved by the Animal Ethics Committee of Fujian Medical University.

All animals were first acclimatized to run on a treadmill at a speed of 10 m/min for 30 min/day for 1 week. Subsequently, animals in the LIR, MIR, and HIR groups exercised regularly according to the previously described running protocols for 1, 4, and 8 weeks (Ni et al., 2013). Training speed and inclination varied as follows: LIR, 15.2 m/min with 0° incline for 60 min, 5 days/week; MIR, 19.3 m/min with 5° incline for 60 min, 5 days/week; and HIR, 26.8 m/min with 10° incline for 60 min, 5 days/week. The exercise-trained rats were encouraged to run by mild electrical stimulation. Meanwhile, the rats in the SED group maintained sedentary lifestyles.

### Echocardiography

Transthoracic echocardiography was performed for all rats 24 h after the last bout of exercise, and heart function was assessed by echocardiography (Vivid E9, General Electric Company, CT, United States). The rats were anesthetized using 10% chloral hydrate (3 mL/kg intraperitoneally). The following structural variables were measured according to M-mode tracings: LV end-diastolic dimension (LVEDD), LV end-systolic dimension (LVESD), LV end-diastolic volume (LVEDV), LV end-systolic volume (LVESV), LV posterior wall thickness (LVPWT), interventricular septum thickness (IVST), ejection fraction (EF), fractional shortening (FS), stroke volume (SV), heart rate (HR), and cardiac output (CO).

### Biochemical Measurements

After completion of the transthoracic echocardiogram, the abdominal cavity was quickly opened, and blood samples were collected from the ventral aorta. The blood samples were kept at room temperature for 1 h, centrifuged at 300 × g for 15 min, and stored at −80°C until analysis. Cardiac troponin T (cTnI) was measured by enzyme-linked immunosorbent assay (ELISA) according to the manufacturer’s instructions (Elabscience Biotechnology Co., Ltd., Wuhan, China).

### Histological Evaluation

After completing echocardiography, the whole hearts were perfused with 250–300 mL normal saline, rapidly excised, and weighed on an electronic balance to calculate the heart mass (HM) to body mass (BM) ratio (HM/BM). Subsequently, the hearts were either fixed in 4% paraformaldehyde for histological analysis or frozen in liquid nitrogen for protein analysis. After 24 h fixation in paraformaldehyde solution, the atria were removed, and the ventricles were dehydrated with ethanol, embedded in paraffin, and cut transversely into 5 μm sections. Hematoxylin-eosin (HE) staining and wheat germ agglutinin were performed on the heart sections to measure myocyte cross-sectional area. Picrosirius red (PSR) staining was performed to define the average volume of collagen deposition in the heart. The captured images were analyzed using Image-Pro Plus 6.0 (Media Cybernetics, Inc., Rockville, MD, United States).

### Western Blot Analysis

Proteins were extracted from snap-frozen heart tissue using a RIPA buffer (P0013B; Beyotime Biotechnology). A bicinchoninic acid (BCA) protein analysis kit (P0010S; Beyotime Biotechnology) was used to quantify the amount of protein, and then the protein concentrations were normalized before all Western blot experiments. Equal amounts of protein were separated by 10% sodium dodecyl sulfate polyacrylamide gel electrophoresis (SDS-PAGE) and transferred onto a nitrocellulose membrane. Subsequently, these membranes were blocked with non-fat milk for 1 h at room temperature, followed by overnight incubation at 4°C with primary antibodies against MEK (ab178876, 1:20,000; Abcam), ERK (ab36991, 1:2,000; Abcam), p-ERK (Thr202/Tyr204) (#4376, 1:1,000; CST), and GAPDH (60004-1-Ig, 1:2,000; Proteintech). The membranes were then incubated with horseradish peroxidase-conjugated secondary antibodies (SA00001-1, 1:20,000; Proteintech) for 1 h at room temperature. The membranes were developed with chemiluminescence reagents (P0019; Beyotime Biotechnology), the chemiluminescence signals were analyzed using an Azure Biosystems C600 imager (Azure Biosystems Inc., CA, United States).

### Statistical Analysis

All statistical analyses were carried out using SPSS 23.0 software (IBM Corp., Armonk, NY, United States). Data are expressed as mean ± standard deviation. Differences between multiple groups were tested by one-way analysis of variance (ANOVA). *Post hoc* LSD or Kruskal–Wallis H tests were used for multiple comparisons. A *p*-value of under 0.05 was considered statistically significant.

## Results

### Body Weight and Heart Weight

**FIGURE 1:**
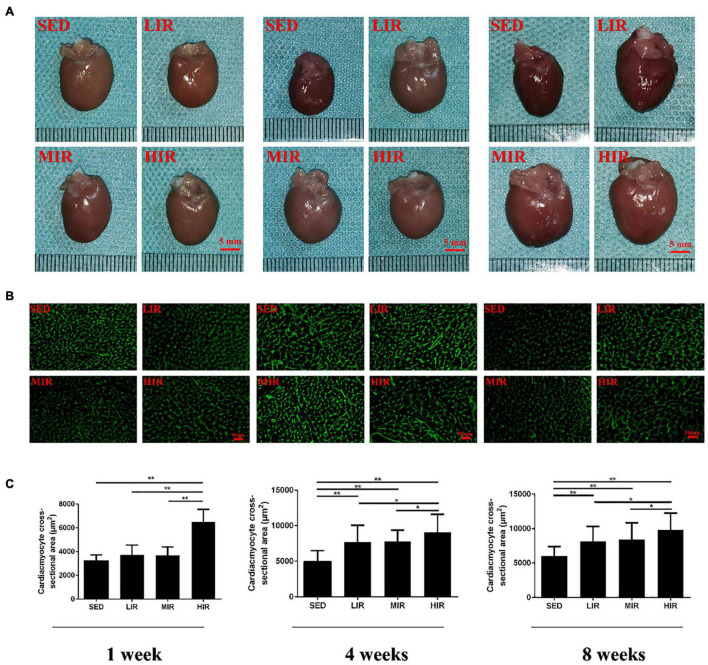
SD rats were subjected to different treadmill running intensities for 1, 4, and 8 weeks. **(A–C)** Hypertrophic changes of the hearts were observed by gross morphologic examination **(A)** and WGA staining **(B,C)**. Scale bar: 5 mm **(A)**, 50 μm **(B)**. Data are presented as the means ± SEM. *P < 0.05, **P < 0.01.

We expected different exercise intensities to be associated with cardiac hypertrophy. At the gross level, the hearts of the exercised rats were remarkably larger than those of the SED animals and presented with typical hypertrophic changes after 4 and 8 weeks of exercise (Figure 1A). The data in Table 1 show that there was a significant decrease in body weight in the MIR and HIR groups after 1, 4, and 8 weeks of exercise and a lower body weight in the LIR group compared with the SED group after 8 weeks of exercise (*p* < 0.05). A significant difference in heart weight was identified in the MIR and HIR groups at 8 weeks of exercise (*p* < 0.05). When comparing the HM/BM ratios, although 4 and 8 weeks of different treadmill running intensities resulted in a significant increase (*p* < 0.05), no significant differences were found between the SED group and other exercise groups after 1 week, which was consistent with the gross morphologic examination. At the tissue level, we found that the HIR group displayed significantly greater ventricular myocyte size compared with the SED group, and this also held true between the HIR group and other exercise groups after 1 week of exercise (Figures 1B,C). When compared with the ventricular myocytes of the SED group, the cross-sectional area of the ventricular myocytes of the rats subjected to different treadmill running intensities for 4 and 8 weeks was significantly larger (*p* < 0.01).

**TABLE 1:** Average body weight and heart weight along with heart weight to body weight ratio for the different groups studied (*n* = 6 in each case).

	SED	LIR	MIR	HIR
	1 week			
BW (g)	326.5 ± 15	323.3 ± 11.9	304.5 ± 15.3^a^	294.8 ± 20.8^b^^d^
HW (mg)	1039 ± 46	1055.2 ± 100.3	986 ± 82.8	982.4 ± 82.5
HW/BW (mg/g)	3.2 ± 0.2	3.3 ± 0.2	3.2 ± 0.3	3.3 ± 0.1
	4 weeks			
BW (g)	435 ± 25.5	405.5 ± 25.6	384.2 ± 36.2^b^	368.3 ± 24.9^b^
HW (mg)	1126.5 ± 127.1	1233.8 ± 85.1	1247.2 ± 134.4	1281.5 ± 130.7
HW/BW (mg/g)	2.6 ± 0.1	3.1 ± 0.3^b^	3.3 ± 0.3^b^	3.5 ± 0.2^b^^c^
	8 weeks			
BW (g)	479.8 ± 27.4	448.2 ± 29.8^a^	419.2 ± 15.4^b^	407.3 ± 17.2^b^^c^
HW (mg)	1192.8 ± 87.8	1192.8 ± 87.8	1351.2 ± 106.7^a^	1398.7 ± 121.5^b^
HW/BW (mg/g)	2.5 ± 0.1	2.9 ± 0.1^b^	3.2 ± 0.2^b^^c^	3.4 ± 0.2^b^^d^

*BW body weight, HW heart weight, HW/BW heart weight/body weight ratio.*

*Data are presented as means ± SEM.*

*^a^P < 0.05 vs. SED.*

*^b^P < 0.01 vs. SED.*

*^c^P < 0.05 vs. LIR.*

*^d^P < 0.01 vs. LIR. (at the same age).*

### Morphometry and Histology

**FIGURE 2:**
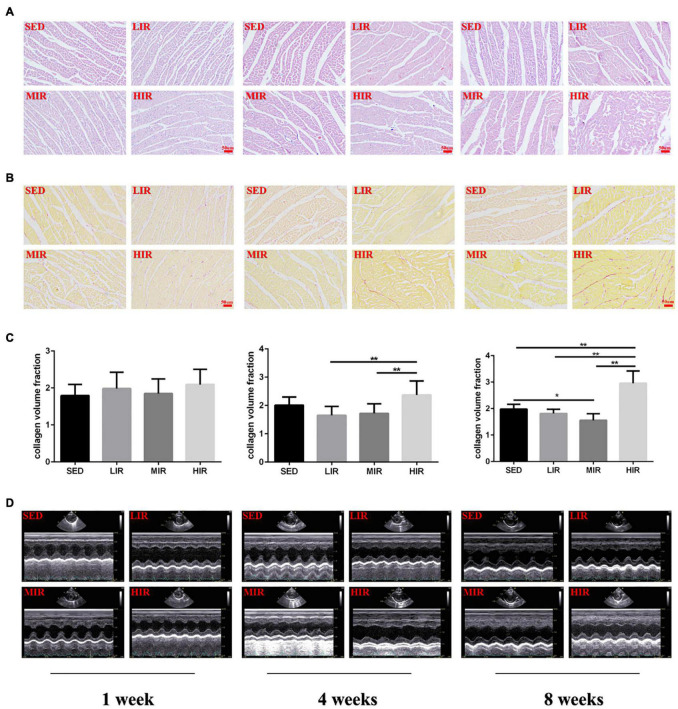
SD rats were subjected to different treadmill running intensities for 1, 4, and 8 weeks. **(A–C)** Histological changes of the hearts were observed by HE staining **(A)**, PSR staining **(B,C)**. Scale bar: 50 μm **(A,B)**. **(D)** typical M-mode images of echocardiogram in the left ventricle. Data are presented as the means ± SEM. *P < 0.05, **P < 0.01.

Myocardial structural changes and fibrosis are hallmarks of cardiac hypertrophic remodeling. Compared with the myocardial structure of the SED group, the cardiomyocytes in the HIR group began to occasionally and slightly rupture after 4 weeks of exercise, while those after 8 weeks of exercise were irregular in shape and disorganized in arrangement (Figure 2A). Similar to the changes in myocardial structure, collagen deposition in the myocardium was also detected in the HIR group following 4 and 8 weeks of exercise, showing a significant increase compared with the SED group and other exercise groups (*p* < 0.01; Figures 2B,C). However, the fibrosis in the ventricular myocytes decreased significantly in the MIR group after 8 weeks (*p* < 0.05). After 1 week, all trained rats showed no changes in myocardial structure and no collagen deposition in the myocardium.

### Morphological and Functional Parameters

To determine whether cardiac hypertrophy caused by exercises of varying intensities differed in terms of morphology and function, we analyzed the rats’ heart using echocardiography (Figure 2D). The data in Table 2 show that LVEDD, LVESD, LVEDV, and LVESV were significantly increased in the LIR and MIR groups compared with the SED group after 1 week, but there were no significant changes in the left ventricle internal diameter or volumes in the HIR group. However, the resting cardiac function in rats as assessed by FS and EF was significantly lower in the LIR and MIR groups. After 4 weeks of exercise, the data in Table 3 revealed that MIR decreased LVEDV and increased LVESD and LVESV (all *p* < 0.05). Moreover, the decrease in LVEDV and HR resulted in lower CO in the HIR group compared with the LIR and MIR groups. The data in Table 4 showed that 8-week low-to-medium-intensity running resulted in a significant increase in LVEDD compared with the SED group (*p* < 0.05). Additionally, the HR in the LIR group was significantly higher than that of the SED group, resulting in a significant increase in CO (*p* < 0.05).

**TABLE 2:** Echocardiographic analysis of SD rats subjected to different treadmill running intensities for 1 week.

Criteria	SED	LIR	MIR	HIR
LVEDD (mm)	6.493 ± 0.979	7.495 ± 0.48^a^	7.628 ± 0.677^a^	6.8 ± 0.456
LVESD (mm)	3.07 ± 0.814	4.46 ± 0.576^b^	4.285 ± 0.701^b^	3.602 ± 0.538^c^
LVEDV (lL)	0.657 ± 0.222	0.942 ± 0.168^a^	0.998 ± 0.219^b^	0.72 ± 0.142^d^
LVESV (lL)	0.085 ± 0.062	0.225 ± 0.079^b^	0.207 ± 0.089^a^	0.124 ± 0.054^c^
SV (lL)	0.57 ± 0.186	0.717 ± 0.129	0.82 ± 0.206^a^	0.626 ± 0.117
IVST (mm)	1.567 ± 0.121	1.533 ± 0.216	1.417 ± 0.147	1.48 ± 0.311
LVPWT (mm)	2 ± 0.237	1.767 ± 0.137^a^	1.75 ± 0.164^a^	1.88 ± 0.217
EF (%)	86.923 ± 4.504	76.211 ± 5.784^b^	79.323 ± 6.731^a^	82.571 ± 6.55
FS (%)	51.733 ± 5.072	38.446 ± 7.29^b^	43.678 ± 7.186^a^	46.719 ± 6.314
HR(bpm)	440.167 ± 31.141	413.5 ± 45.465	405.167 ± 23.609	427.4 ± 25.706
CO	251.675 ± 83.315	276.854 ± 53.919	354.382 ± 30.831^a^	254.583 ± 87.635^d^

*Data are presented as the means ± SEM.*

*^a^P < 0.05 vs. SED.*

*^b^P < 0.01 vs. SED.*

*^c^P < 0.05 vs. LIR.*

*^d^P < 0.05 vs. MIR.*

**TABLE 3:** Echocardiographic analysis of SD rats subjected to different treadmill running intensities after 4 weeks.

Criteria	SED	LIR	MIR	HIR
LVEDD (mm)	6.697 ± 0.847	6.952 ± 0.407	7.552 ± 0.329	6.528 ± 0.712
LVESD (mm)	4.015 ± 0.827	3.614 ± 0.539	4.458 ± 0.508^b^	3.883 ± 0.553
LVEDV (lL)	0.707 ± 0.25	0.766 ± 0.12	0.96 ± 0.113^a^	0.653 ± 0.19
LVESV (lL)	0.175 ± 0.096	0.124 ± 0.047	0.218 ± 0.062^b^	0.153 ± 0.066
SV (lL)	0.532 ± 0.158	0.64 ± 0.9	0.742 ± 0.07	0.5 ± 0.158
IVST (mm)	1.582 ± 0.116	1.512 ± 0.232	1.567 ± 0.186	1.4 ± 0.237
LVPWT (mm)	1.903 ± 0.152	1.86 ± 0.163	1.933 ± 0.121	1.783 ± 0.232
EF(%)	78.347 ± 4.861	83.461 ± 5.951	76.589 ± 6.245	75.89 ± 7.862
FS(%)	42.306 ± 4.308	47.809 ± 6.952	40.696 ± 5.733	40.209 ± 6.849
HR(bpm)	383 ± 39.542	411.2 ± 42.222	360.833 ± 56.926	341 ± 71.828^b^
CO	205.042 ± 70.87	263.764 ± 46.099	266.84 ± 44.624	166.91 ± 54.841^b^^c^

*Data are presented as the means ± SEM.*

*^a^P < 0.05 vs. SED.*

*^b^P < 0.05 vs. LIR.*

*^c^P < 0.01 vs. MIR.*

**TABLE 4:** Echocardiographic analysis of SD rats subjected to different treadmill running intensities after 8 weeks.

Criteria	SED	LIR	MIR	HIR
LVEDD (mm)	6.458 ± 0.761	7.117 ± 0.624^a^	7.202 ± 0.635^a^	7.065 ± 0.618
LVESD (mm)	3.197 ± 0.849	3.72 ± 0.952	4.128 ± 0.724	3.993 ± 0.722
LVEDV (lL)	0.64 ± 0.187	0.828 ± 0.203	0.85 ± 0.205	0.805 ± 0.201
LVESV (lL)	0.097 ± 0.079	0.15 ± 0.118	0.186 ± 0.088	0.17 ± 0.086
SV (lL)	0.498 ± 0.14	0.68 ± 0.121^a^	0.664 ± 0.144	0.635 ± 0.127
IVST (mm)	1.508 ± 0.165	1.643 ± 0.074	1.512 ± 0.223	1.637 ± 0.181
LVPWT (mm)	2.01 ± 0.222	1.998 ± 0.085	1.856 ± 0.134	1.995 ± 0.157
EF (%)	85.355 ± 7.926	82.997 ± 8.031	79.036 ± 6.454	79.405 ± 6.301
FS (%)	50.387 ± 8.344	47.633 ± 8.177	43.156 ± 6.244	43.2 ± 5.843
HR (bpm)	365.833 ± 61.029	421.5 ± 43.284^a^	410.4 ± 28.676	400 ± 23.401
CO	203.713 ± 69.378	288.195 ± 67.943^a^	272.106 ± 63.302	253.157 ± 46.626

*Data are presented as the means ± SEM.*

*^a^P < 0.05 vs. SED.*

### Serum Cardiac Troponin I Levels

**FIGURE 3:**
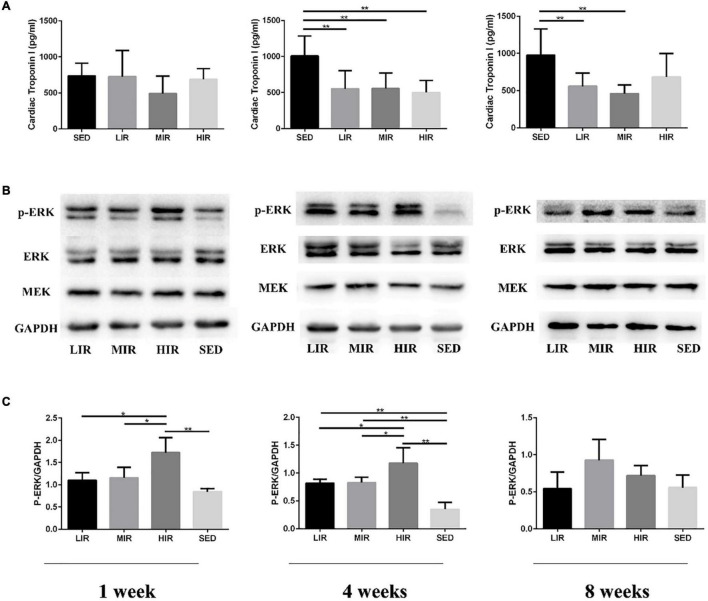
Effects of different treadmill running intensities after 1, 4, and 8 weeks on serum cTnI levels **(A)** and ERK phosphorylation **(B,C)** in rats. Data are presented as the means ± SEM. *P < 0.05, **P < 0.01.

Figure 3A shows the levels of the serum cTnI of the different treadmill running intensity groups. Compared with the SED group, no significant difference was found in cTnI content in the exercise groups with varying intensities following 1 week of exercise. However, after 4 weeks of exercise, the levels of cTnI in the LIR, MIR, and HIR groups were significantly lower than those of the SED group. After 8 weeks of exercise, the levels of cTnI in the LIR and MIR groups were significantly lower than those of the SED group, whereas there was no significant difference between the HIR and SED groups.

### Protein Analysis

We assessed MEK-ERK1/2 signaling to investigate the mechanisms underlying the differences in exercise-induced hypertrophy. As shown in Figures 3B,C, after 1 week of exercise, the phosphorylation of ERK1/2 was significantly increased in the HIR group compared with the SED group (*p* < 0.01), as well as the other exercise groups (*p* < 0.05), whereas there was no difference between the LIR, MIR, and SED groups. After 4 weeks of exercise, there was a significant increase in the phosphorylation of ERK1/2 in the LIR, MIR, and HIR groups compared with the SED group (*p* < 0.01). However, after 8 weeks of exercise, there were no longer any significant differences between the groups.

## Discussion

In the present study, we provided an *in vivo* structural and functional comparison of exercise-induced hypertrophy in a rat model, depicting that changes in cardiac response differ based on exercise intensity and potential signal transduction. Here, our findings indicate that running leads to cardiac hypertrophy in an intensity-dependent manner. In contrast to LIR and MIR, 8 weeks of HIR-induced cardiac hypertrophy was characterized by potential cardiomyocyte injury, which increased the risk of pathological development. Furthermore, the ERK signaling pathway was mainly involved in the compensatory hypertrophic process of the myocardium in the early stage of exercise and was positively correlated with exercise load. However, long-term exercise may attenuate ERK signaling activation.

In the process of long-term adaptation to regular exercise, myocardial hypertrophy leads to an increased blood pumping ability to meet the increased metabolic needs of the whole body (Ellison et al., 2012). Cardiac hypertrophy is characterized by myocyte hypertrophy, structural rearrangement, and the accumulation of cardiac collagen (Krzesiak et al., 2017). Following exercise training in humans and animal models, heart mass typically increases (Wang et al., 2010; Davos, 2019). Macroscopically, we found that the increases in the size of the hearts in the running groups were more obvious after 4 and 8 weeks. Moreover, the HM/BM ratio, which is widely used to assess cardiac hypertrophy, has shown similar results. Although the ratio of heart mass to tibia length (HM/TL) would seem to be more reliable, data from other studies (Shioi et al., 2003; Tang et al., 2017) have indicated that there were no significant differences between HM/BM and HM/TL. Microscopically, our study showed that 1 week of HIR induced cardiac hypertrophy, while myocyte hypertrophy mirrored the macroscopic situation only after 4 and 8 weeks of running. Indeed, there is evidence that high-intensity treadmill models more easily alter cardiac phenotypes in rodents (Kemi et al., 2005; Liao et al., 2015). Furthermore, these cardiac dimensions are consistent with previous experimental results (Tang et al., 2011), which have suggested that myocardial hypertrophy occurs in tandem with an increase in exercise intensity and extension of exercise time. To induce a hypertrophic heart, a longer period of exercise may be required.

In terms of structural arrangement and cardiac collagen, we found that low-to-medium-intensity running led to proper cell morphological characteristics and decreased fibrosis, which likely contributed to overall myocardial tissue integrity and homeostasis for cardiac beneficial effects (Libonati et al., 2011; Pagan et al., 2019). HIR, however, may have a different impact on the heart. Although few studies have compared the effects of different exercise intensities on histological changes in rat hearts, Liao et al. (2015) have shown that a wide range of largely primary myocellular defects and myocardium ischemia were observed after high-intensity exercise. This is consistent with our finding of some counterproductive cardiac consequences induced by exercise. We also noted that collagen deposition in the myocardium also increased significantly. An elegant study demonstrated increased cardiac fibrosis after long-term intensive treadmill running in a rat model and represented potentially adverse cardiac remodeling (Benito et al., 2011). Data from reviews also suggest the development of ventricular fibrosis as assessed by magnetic resonance imaging (MRI) in endurance athletes (Malek and Bucciarelli-Ducci, 2020). As a result of the accumulation of these minor injuries, exercise may produce unfavorable results.

The mechanisms by which high-intensity exercise promotes cardiac pathologies are unknown. It is possible that long-term cardiac overload plays a role by promoting physiological remodeling in the early phases, but the heart, as the circulation center of energy and nutrition, is very sensitive to ischemia and hypoxia, which may eventually become maladaptive in the long term. A recent study supports the idea that balanced intense exercise training induces hypertrophy that is not associated with pathological remodeling (Olah et al., 2021).

Transthoracic echocardiography is accurate and widely used in the field to assess cardiac structure and function. The present study found that low-to-medium intensity running induced a significant increase in absolute LV diastolic diameter values and a decrease in LV relative wall thickness after 1 week. These results indicate a shift to eccentric LV remodeling. According to Morganroth’s dichotomous concept, regular aerobic sports, such as running, are accompanied by cardiac chamber dilation, which is referred to as eccentric hypertrophy (Morganroth et al., 1975; Ellison et al., 2012). However, in contrast, other studies have demonstrated that short-duration exercise appears to have little in the way of a negative impact on ventricular function (Oxborough et al., 2010). Our study also showed that FS and EF significantly decreased in the LIR and MIR groups, but not in the HIR group, in the short term. This disparity might be a consequence of myocyte hypertrophy in the HIR group. In short-term low-to-moderate-intensity training, cardiomyocyte contractility and maximal oxygen consumption (VO_2_max) were like those of the control group (Kemi et al., 2004). Meanwhile, heart growth is related to an increase in chamber dilation. In order to maintain the normal ratio of the number of cardiomyocytes to blood capillaries, there may be a temporary decline in systolic function. Moreover, the impact of prolonged running on EF and FS was not significantly altered between the exercise groups and the SED group after weeks 4 and 8. The observed unchanged cardiac function is in line with recent findings on rat and athlete hearts (Oosthuyse et al., 2012; Asif et al., 2018). Although transthoracic echocardiography is accurate and widely used to evaluate cardiac structure and function in this field, MRI technology provides more accurate and reproducible measurements of cardiac function (Nikolaidou and Karamitsos, 2020; Russo et al., 2020).

cTnI is a highly sensitive and specific marker of myocardial injury and is suitable for early and late diagnoses (Mair et al., 1996). In the present study, no obvious myocardial injury was observed after treadmill running with varying intensities. In contrast to previous observations of animal models (Liao et al., 2015) and a large number of human exercise studies (Kosowski et al., 2019), the release of cTnI was markedly decreased after 4 weeks of running, and the same state also occurred after 8 weeks for the LIR and MIR groups, indicating that the leakage of cTnI decreased after exercise. The reason for this situation is that cardiac biomarker concentrations tend to return to baseline at 24–48 h postexercise (Lippi and Banfi, 2010). However, according to the current data, running for 8 weeks between the HIR and SED groups has no obvious changes, which may lead to potential myocardial injury. Our HE and PSR staining of the myocardium showed signs of sporadic cardiomyocyte damage and increased fibrosis. However, this probably represents a standard exercise intensity-dependent response rather than a pathological response.

It is generally accepted that ERK1/2 activation is essential for cardiac hypertrophy (Gallo et al., 2019). However, several partially contradictory studies have indicated that ERK1/2 can lead not only to maladaptive cardiac hypertrophy (Lorenz et al., 2009; Tomasovic et al., 2020), but also physiological hypertrophy (Bueno et al., 2000; Mutlak et al., 2018), and some studies have also suggested that it has no effect on cardiac hypertrophy (Purcell et al., 2007). In addition, Kehat et al. (2011) demonstrated that the ERK1/2 signaling pathway uniquely regulates the balance between eccentric and concentric growth of the heart. It has been theorized that the model used, circumstances, and upstream signals may affect the outcomes of these mouse studies on ERK1/2-mediated cardiac hypertrophy. In our rat model, we found that only HIR resulted in the activation of ERK1/2 after 1 week. The amount of phosphorylated ERK1/2 increased significantly by 134.3, 137.1, and 237.1% when the rats ran at low, medium, and high intensities, respectively, for 4 weeks. However, there were no significant changes in ERK1/2 activation between the running groups of varying intensities and the SED group at week 8. As reported, exercise induces the activation of multiple mitogen-active protein kinase (MAPK) pathways in the heart, an effect that gradually declines with the development of exercise-induced cardiac hypertrophy (Iemitsu et al., 2006). Interestingly, Hayashida et al. (2001) demonstrated that c-Jun N-terminal kinases (JNK) and p38-MAPK were similarly differentially regulated in the stages of the chronic hypertrophic process, while angiotensin (Ang) II-induced ERK activation was preserved. Therefore, the combined results from previous studies and our present evidence suggested that ERK1/2 represents a positive regulator in the progression of cardiac hypertrophy and is correlated with running intensity, which is, however, alleviated by increased running duration. Our research may provide valuable input regarding striking a balance between intensity and duration in terms of exercise-induced cardiac hypertrophy.

In summary, we confirmed that the effect of running on cardiac hypertrophy is intensity dependent. Compared with LIR and MIR, myocardial hypertrophy induced by HIR at 8 weeks was characterized by sporadic heart injuries, cumulative fibrosis, and increased myocardial enzymes, increasing the risk of pathological development. In addition, the ERK signaling pathway was mainly involved in the compensatory hypertrophy process of early exercise and was positively correlated with exercise load. However, long-term exercise may weaken the activation of the ERK signal.

### Study Limitations

There are several potential limitations of the current study that need to be acknowledged. Firstly, this study did not consider gender differences when examining the impact of physiological differences on exercise. A model involving male rats was part of our devised projects. In the future, another individualized protocol for female rats warrants further investigation. Secondly, although we applied the previous exercise regimens to differentiate running loading, measurements of VO_2_max might better evaluate the real cardiac capability of different intensities, in addition to running distance, slope, and speed. The possible influence of running intensity should therefore be assessed in future studies. Lastly, our data on the decreased cardiac parameters of EF and FS suggest the initiation of ventricular function degradation. However, the impact of the observed functional changes in the short term remains unanswered. Further detection methods, such as MRI, are needed to clarify the possibility of myocardial dysfunction described in the present study.

## Data Availability Statement

The original contributions presented in the study are included in the article/supplementary material, further inquiries can be directed to the corresponding author/s.

## Ethics Statement

The animal study was reviewed and approved by the Animal Ethics Committee of Fujian Medical University.

## Author Contributions

ZY and GN conceived research concept and drafted, revised, and edited the manuscript. ZY and NZ designed and performed the experiments, analyzed the data, and prepared the figures. NZ, JL, and TL contributed to the sample collection and sample storage. The authors read and approved the final manuscript. All authors contributed to the article and approved the submitted version.

## Conflict of Interest

The authors declare that the research was conducted in the absence of any commercial or financial relationships that could be construed as a potential conflict of interest.

## Publisher’s Note

All claims expressed in this article are solely those of the authors and do not necessarily represent those of their affiliated organizations, or those of the publisher, the editors and the reviewers. Any product that may be evaluated in this article, or claim that may be made by its manufacturer, is not guaranteed or endorsed by the publisher.

## References

[B1] Asif Y. Wlodek M. E. Black M. J. Russell A. P. Soeding P. F. Wadley G. D. (2018). Sustained cardiac programming by short-term juvenile exercise training in male rats. *J. Physiol.* 596 163–180. 10.1113/JP275339 29143975 PMC5767703

[B2] Benito B. Gay-Jordi G. Serrano-Mollar A. Guasch E. Shi Y. Tardif J. C. (2011). Cardiac arrhythmogenic remodeling in a rat model of long-term intensive exercise training. *Circulation* 123 13–22. 21173356 10.1161/CIRCULATIONAHA.110.938282

[B3] Bueno O. F. De Windt L. J. Tymitz K. M. Witt S. A. Kimball T. R. Klevitsky R. (2000). The MEK1-ERK1/2 signaling pathway promotes compensated cardiac hypertrophy in transgenic mice. *EMBO J.* 19 6341–6350. 10.1093/emboj/19.23.6341 11101507 PMC305855

[B4] Davos C. H. (2019). Do we have to reconsider the guidelines for exercise intensity determination in cardiovascular rehabilitation? *Eur. J. Prev. Cardiol.* 26 1918–1920. 31446786 10.1177/2047487319871870

[B5] Ellison G. M. Waring C. D. Vicinanza C. Torella D. (2012). Physiological cardiac remodelling in response to endurance exercise training: cellular and molecular mechanisms. *Heart* 98 5–10. 10.1136/heartjnl-2011-300639 21880653

[B6] Gallo S. Vitacolonna A. Bonzano A. Comoglio P. Crepaldi T. (2019). ERK: a key player in the pathophysiology of cardiac hypertrophy. *Int. J. Mol. Sci.* 20:2164. 10.3390/ijms20092164 31052420 PMC6539093

[B7] Hayashida W. Kihara Y. Yasaka A. Inagaki K. Iwanaga Y. Sasayama S. (2001). Stage-specific differential activation of mitogen-activated protein kinases in hypertrophied and failing rat hearts. *J. Mol. Cell Cardiol.* 33 733–744. 10.1006/jmcc.2001.1341 11273726

[B8] Iemitsu M. Maeda S. Jesmin S. Otsuki T. Kasuya Y. Miyauchi T. (2006). Activation pattern of MAPK signaling in the hearts of trained and untrained rats following a single bout of exercise. *J. Appl. Physiol.* 101 151–163. 10.1152/japplphysiol.00392.2005 16484365

[B9] Kehat I. Davis J. Tiburcy M. Accornero F. Saba-El-Leil M. K. Maillet M. (2011). Extracellular signal-regulated kinases 1 and 2 regulate the balance between eccentric and concentric cardiac growth. *Circ. Res.* 108 176–183. 10.1161/CIRCRESAHA.110.231514 21127295 PMC3032171

[B10] Kemi O. J. Haram P. M. Loennechen J. P. Osnes J. B. Skomedal T. Wisloff U. (2005). Moderate vs. high exercise intensity: differential effects on aerobic fitness, cardiomyocyte contractility, and endothelial function. *Cardiovasc. Res.* 67 161–172. 10.1016/j.cardiores.2005.03.010 15949480

[B11] Kemi O. J. Haram P. M. Wisløff U. Ellingsen Ø (2004). Aerobic fitness is associated with cardiomyocyte contractile capacity and endothelial function in exercise training and detraining. *Circulation* 109 2897–2904. 10.1161/01.CIR.0000129308.04757.72 15173028

[B12] Khoury S. R. Evans N. S. Ratchford E. V. (2019). Exercise as medicine. *Vasc. Med.* 24 371–374. 31144595 10.1177/1358863X19850316

[B13] Konhilas J. P. Maass A. H. Luckey S. W. Stauffer B. L. Olson E. N. Leinwand L. A. (2004). Sex modifies exercise and cardiac adaptation in mice. *Am. J. Physiol. Heart Circ. Physiol.* 287 H2768–H2776. 15319208 10.1152/ajpheart.00292.2004 PMC2637113

[B14] Kosowski M. Mlynarska K. Chmura J. Kustrzycka-Kratochwil D. Sukiennik-Kujawa M. Todd J. A. (2019). Cardiovascular stress biomarker assessment of middle-aged non-athlete marathon runners. *Eur. J. Prev. Cardiol.* 26 318–327. 10.1177/2047487318819198 30744458

[B15] Krzesiak A. Delpech N. Sebille S. Cognard C. Chatelier A. (2017). Structural, contractile and electrophysiological adaptations of cardiomyocytes to chronic exercise. *Adv. Exp. Med. Biol.* 999 75–90. 10.1007/978-981-10-4307-9_5 29022258

[B16] Liao J. Li Y. Zeng F. Wu Y. (2015). Regulation of mTOR pathway in exercise-induced cardiac hypertrophy. *Int. J. Sports Med.* 36 343–350. 25607521 10.1055/s-0034-1395585

[B17] Libonati J. R. Sabri A. Xiao C. Macdonnell S. M. Renna B. F. (2011). Exercise training improves systolic function in hypertensive myocardium. *J. Appl. Physiol.* 111 1637–1643. 21921241 10.1152/japplphysiol.00292.2011 PMC3233879

[B18] Lippi G. Banfi G. (2010). Exercise-related increase of cardiac troponin release in sports: an apparent paradox finally elucidated? *Clin. Chim. Acta Int. J. Clin. Chem.* 411 610–611. 10.1016/j.cca.2010.01.009 20079724

[B19] Ljones K. Ness H. O. Solvang-Garten K. Gaustad S. E. Hoydal M. A. (2017). Acute exhaustive aerobic exercise training impair cardiomyocyte function and calcium handling in Sprague-Dawley rats. *PLoS One* 12:e0173449. 10.1371/journal.pone.0173449 28273177 PMC5342256

[B20] Lorenz K. Schmitt J. P. Schmitteckert E. M. Lohse M. J. (2009). A new type of ERK1/2 autophosphorylation causes cardiac hypertrophy. *Nat. Med.* 15 75–83. 10.1038/nm.1893 19060905

[B21] Mair J. Genser N. Morandell D. Maier J. Mair P. Lechleitner P. (1996). Cardiac troponin I in the diagnosis of myocardial injury and infarction. *Clin. Chim. Acta Int. J. Clin. Chem.* 245 19–38. 10.1016/0009-8981(95)06168-1 8646813

[B22] Malek L. A. Bucciarelli-Ducci C. (2020). Myocardial fibrosis in athletes-Current perspective. *Clin. Cardiol.* 43 882–888. 10.1002/clc.23360 32189357 PMC7403702

[B23] Mi C. Qin X. Hou Z. Gao F. (2019). Moderate-intensity exercise allows enhanced protection against oxidative stress-induced cardiac dysfunction in spontaneously hypertensive rats. *Braz. J. Med. Biol. Res.* 52:e8009. 10.1590/1414-431X20198009 31116256 PMC6526746

[B24] Moreira J. B. N. Wohlwend M. Wisløff U. (2020). Exercise and cardiac health: physiological and molecular insights. *Nat. Metab.* 2 829–839. 32807982 10.1038/s42255-020-0262-1

[B25] Morganroth J. Maron B. J. Henry W. L. Epstein S. E. (1975). Comparative left ventricular dimensions in trained athletes. *Ann. Intern. Med.* 82 521–524. 1119766 10.7326/0003-4819-82-4-521

[B26] Mutlak M. Schlesinger-Laufer M. Haas T. Shofti R. Ballan N. Lewis Y. E. (2018). Extracellular signal-regulated kinase (ERK) activation preserves cardiac function in pressure overload induced hypertrophy. *Int. J. Cardiol.* 270 204–213. 29857938 10.1016/j.ijcard.2018.05.068

[B27] Nakamura M. Sadoshima J. (2018). Mechanisms of physiological and pathological cardiac hypertrophy. *Nat. Rev. Cardiol.* 15 387–407. 29674714 10.1038/s41569-018-0007-y

[B28] Ni G. X. Liu S. Y. Lei L. Li Z. Zhou Y. Z. Zhan L. Q. (2013). Intensity-dependent effect of treadmill running on knee articular cartilage in a rat model. *BioMed Res. Int.* 2013:172392. 10.1155/2013/172392 24693534 PMC3892754

[B29] Nikolaidou C. Karamitsos T. (2020). Should everyone have an MRI in heart failure? *Cardiovasc. Diagn. Ther.* 10 549–553. 32695635 10.21037/cdt.2019.12.06 PMC7369283

[B30] Olah A. Barta B. A. Sayour A. A. Ruppert M. Virag-Tulassay E. Novak J. (2021). Balanced intense exercise training induces atrial oxidative stress counterbalanced by the antioxidant system and atrial hypertrophy that is not associated with pathological remodeling or arrhythmogenicity. *Antioxidants* 10:452. 33803975 10.3390/antiox10030452 PMC7999710

[B31] Olah A. Nemeth B. T. Matyas C. Horvath E. M. Hidi L. Birtalan E. (2015). Cardiac effects of acute exhaustive exercise in a rat model. *Int. J. Cardiol.* 182 258–266. 10.1016/j.ijcard.2014.12.045 25585360

[B32] Oosthuyse T. Avidon I. Likuwa I. Woodiwiss A. J. (2012). Progression of changes in left ventricular function during four days of simulated multi-stage cycling. *Eur. J. Appl. Physiol.* 112 2243–2255. 10.1007/s00421-011-2201-z 21997679

[B33] Oxborough D. Birch K. Shave R. George K. (2010). “Exercise-induced cardiac fatigue”–a review of the echocardiographic literature. *Echocardiography* 27 1130–1140. 10.1111/j.1540-8175.2010.01251.x 20678128

[B34] Pagan L. U. Damatto R. L. Gomes M. J. Lima A. R. R. Cezar M. D. M. Damatto F. C. (2019). Low-intensity aerobic exercise improves cardiac remodelling of adult spontaneously hypertensive rats. *J. Cell Mol. Med.* 23 6504–6507. 10.1111/jcmm.14530 31317657 PMC6714166

[B35] Purcell N. H. Wilkins B. J. York A. Saba-El-Leil M. K. Meloche S. Robbins J. (2007). Genetic inhibition of cardiac ERK1/2 promotes stress-induced apoptosis and heart failure but has no effect on hypertrophy in vivo. *Proc. Natl. Acad. Sci. U.S.A.* 104 14074–14079. 10.1073/pnas.0610906104 17709754 PMC1955824

[B36] Radovits T. Olah A. Lux A. Nemeth B. T. Hidi L. Birtalan E. (2013). Rat model of exercise-induced cardiac hypertrophy: hemodynamic characterization using left ventricular pressure-volume analysis. *Am. J. Physiol. Heart Circ. Physiol.* 305 H124–H134. 23645462 10.1152/ajpheart.00108.2013

[B37] Russo V. Lovato L. Ligabue G. (2020). Cardiac MRI: technical basis. *Radiol. Med.* 125 1040–1055. 32939626 10.1007/s11547-020-01282-z

[B38] Shioi T. McMullen J. R. Tarnavski O. Converso K. Sherwood M. C. Manning W. J. (2003). Rapamycin attenuates load-induced cardiac hypertrophy in mice. *Circulation* 107 1664–1670. 12668503 10.1161/01.CIR.0000057979.36322.88

[B39] Swain D. P. Franklin B. A. (2006). Comparison of cardioprotective benefits of vigorous versus moderate intensity aerobic exercise. *Am. J. Cardiol.* 97 141–147. 10.1016/j.amjcard.2005.07.130 16377300

[B40] Tang X. Chen X. F. Wang N. Y. Wang X. M. Liang S. T. Zheng W. (2017). SIRT2 acts as a cardioprotective deacetylase in pathological cardiac hypertrophy. *Circulation* 136 2051–2067. 10.1161/CIRCULATIONAHA.117.028728 28947430 PMC5698109

[B41] Tang X. Y. Hong H. S. Chen L. L. Lin X. H. Lin J. H. Lin Z. (2011). Effects of exercise of different intensities on the angiogenesis, infarct healing, and function of the left ventricle in postmyocardial infarction rats. *Coron Artery Dis.* 22 497–506. 10.1097/MCA.0b013e32834993d9 21785345

[B42] Tomasovic A. Brand T. Schanbacher C. Kramer S. Hümmert M. W. Godoy P. (2020). Interference with ERK-dimerization at the nucleocytosolic interface targets pathological ERK1/2 signaling without cardiotoxic side-effects. *Nat. Commun.* 11:1733. 10.1038/s41467-020-15505-4 32265441 PMC7138859

[B43] Wang Y. Wisloff U. Kemi O. J. (2010). Animal models in the study of exercise-induced cardiac hypertrophy. *Physiol. Res.* 59 633–644. 10.33549/physiolres.931928 20406038

[B44] Wasfy M. M. Baggish A. L. (2016). Exercise dose in clinical practice. *Circulation* 133 2297–2313. 27267537 10.1161/CIRCULATIONAHA.116.018093 PMC4902280

[B45] Yan Z. P. Li J. T. Zeng N. Ni G. X. (2021). Role of extracellular signal-regulated kinase 1/2 signaling underlying cardiac hypertrophy. *Cardiol. J.* 28 473–482. 32329039 10.5603/CJ.a2020.0061 PMC8169190

